# Prognosis and NT-proBNP in heart failure patients with preserved versus reduced ejection fraction

**DOI:** 10.1136/heartjnl-2018-314173

**Published:** 2019-04-08

**Authors:** Khibar Salah, Susan Stienen, Yigal M Pinto, Luc W Eurlings, Marco Metra, Antoni Bayes-Genis, Valerio Verdiani, Jan G P Tijssen, Wouter E Kok

**Affiliations:** 1 Heart Centre, Department of Clinical and Experimental Cardiology, Amsterdam Cardiovascular Sciences, Amsterdam UMC, University of Amsterdam, Amasterdam, The Netherlands; 2 Department of Radiology and Nuclear Medicine, Radboud University Medical Centre Nijmegen, Nijmegen, The Netherlands; 3 INSERM, Centre d’Investigation Cliniques Plurithématique, Université de Lorraine, CHRU de Nancy, Nancy, France; 4 Department of Cardiology, VieCuri Medical Centre, Venlo, The Netherlands; 5 Department of Medical and Surgical Specialties, Radiological Sciences and Public Health, Cardiology, University of Brescia, Brescia, Italy; 6 Department of Cardiology, CIBERCV, Hospital Universitari Germans Trias i Pujol, Barcalona, Spain; 7 Department of Internal Medicine and Emergency, Careggi University Hospital, Florence, Italy

**Keywords:** acute decompensated Heart failure, prognosis, preserved left ventricle ejection fraction, NT-proBNP

## Abstract

**Background:**

We assessed the prognostic significance of absolute and percentage change in N-terminal pro-B-type natriuretic peptide (NT-proBNP) levels in patients hospitalised for acute decompensated heart failure with preservedejection fraction (HFpEF) versus heart failure with reduced ejection fraction (HFrEF).

**Methods:**

Patients with left ventricular ejection fraction ≥50% were categorised as HFpEF (n=283), while those with <40% as were categorised as HFrEF (n=776). Prognostic values of absolute and percentage change in NT-proBNP levels for 6 months all-cause mortality after discharge were assessed separately in patients with HFpEF and HFrEF by multivariable adjusted Cox regression analysis. Comorbidities were compared between heart failure groups.

**Results:**

Discharge NT-proBNP levels predicted outcome similarly in HFpEF and HFrEF: for any 2.7-factor increase in NT-proBNP levels, the HR for mortality was 2.14 for HFpEF (95% CI 1.48 to 3.09) and 1.96 for HFrEF (95% CI 1.60 to 2.40). Mortality prediction was equally possible for NT-proBNP reduction of ≤30% (HR 4.60, 95% CI 1.47 to 14.40 and HR 3.36, 95% CI 1.93 to 5.85 for HFpEF and HFrEF, respectively) and for >30%–60% (HR 3.28, 95% CI 1.07 to 10.12 and HR 1.79, 95% CI 0.99 to 3.26, respectively), compared with mortality in the reference groups of >60% reductions in NT-proBNP levels. Prognostically relevant comorbidities were more often present in patients with HFpEF than patients with HFrEF in low (≤3000 pg/mL) but not in high (>3000 pg/mL) NT-proBNP discharge categories.

**Conclusions:**

Our study highlights—after demonstrating that NT-proBNP levels confer the same relative risk information in HFpEF as in HFrEF—the possibility that comorbidities contribute relatively more to prognosis in patients with HFpEF with lower NT-proBNP levels than in patients with HFrEF.

## Introduction

While in-hospital mortality is lower in heart failure with preserved ejection fraction  (HFpEF) than in heart failure patients with reduced left ventricular ejection fraction (HFrEF),^1–3^ mortality after discharge has been reported to be similar to that of patients with HFrEF in studies on patients after hospitalisations for acute decompensated heart failure (ADHF).^3–7^ Recent studies show that in a more stabilised phase, mortality is lower in patients with HFpEF than in patients with HFrEF in populations consisting either of a mix of inpatient and outpatient heart failure patients^8^ or in populations with exclusively outpatients with stable heart failure.^9^ In the latter situation, there are also lower mortality rates compared with the mortality rate after hospitalisations for heart failure.

The prognostic value of absolute levels of B-type natriuretic peptide (BNP) and N-terminal pro-B-type natriuretic peptide (NT-proBNP) has been well established for patients hospitalised for ADHF with either type of heart failure but also specifically for patients with HFpEF.^6 7 10–18^ Prognostic information of a single measurement of natriuretic peptide levels has been specifically investigated in the comparison between patients with HFpEF and HFrEF and has been reported to be equal for the two heart failure types either at admission or at discharge.^6 7 10^ Also, the recently published multicentre study in combined inpatient and outpatient setting with a follow-up of 2 years showed that NT-proBNP levels at clinical stabilisation are strongly and similarly related to survival in heart failure regardless of ejection fraction and that a given level of NT-proBNP portends the same risk of death in HFpEF and HFrEF.^8^

The suggestion that a single baseline or discharge measurement of BNP or NT-proBNP may be equally useful in risk-stratifying patients with ADHF irrespective of the type of heart failure confronts us with the difficulty of explaining why prognosis is similar between the two groups, first of all because natriuretic peptide plasma levels are almost half in HFpEF compared with HFrEF.^6 7 19^ Second, absolute levels of NT-proBNP at admission or at discharge are interpreted as single values, but the assumption may then be that the reduction in NT-proBNP during hospitalisation would be equal in both types of heart failure. A third issue is that the risk assessment of hospitalised heart failure patients with the use of natriuretic peptides is done with relative risks, leaving unexplained that lower discharge natriuretic peptides in patients with HFpEF are associated with similar outcomes as in patients with HFrEF who have higher discharge levels. Finally, even if it can be shown that single values of NT-proBNP are predictive of outcome without distinction between HFpEF and HFrEF, the attainability of these levels may become the factor that determines whether HFpEF or HFrEF have a similar prognosis on a population level.

Therefore, we assessed the prognostic contribution of absolute levels of NT-proBNP and percentage change in NT-proBNP levels in patients with HFpEF and HFrEF hospitalised for ADHF. In addition, we assessed the attainability of several (absolute and relative) discharge NT-proBNP targets in patients with HFpEF and HFrEF. Finally, we assessed the frequencies of prognostically relevant comorbidities in patients with HFpEF and HFrEF for low and high discharge NT-proBNP categories.

## Methods

### Source/study populations

The presently studied population consisted of five of seven cohorts from the *E*uropean co*l*laboration on *a*cute decompe*n*sated *h*eart *f*ailure database with exact data available on left ventricular ejection fraction.^11^ Details on the search strategy, source gathering and explicit information on data collection for these prospective ADHF cohorts have been reported previously.^11^ In addition to these five cohorts, data from the Can NT-*pr*oBNP gu*i*ded therapy during hospital admission for acute decompensated heart failure reduce *m*ortality and re*a*dmissions? (PRIMA II) trial was used for the analyses.^18 20^ The PRIMA II was a randomised controlled trial investigating the effect of NT-proBNP-guided (targeting a >30% NT-proBNP at discharge) versus conventional therapy in patients with ADHF, demonstrating a neutral effect.^18 20 21^

The study population for the present study was assembled by the following criteria: (1) patients were hospitalised because of clinically validated ADHF,^22^ (2) they were discharged alive, (3) left ventricular ejection fraction (LVEF) measurements were performed during admission and (4) NT-proBNP levels were available at admission and/or at discharge. For the present study, patients with heart failure were categorised into three groups: those with LVEF ≥50% were categorised as HFpEF, those with <40% were categorised as HFrEF and patients with LVEF range of 40%–49% were defined as heart failure with midrange EF (HFmrEF), based on the definition of heart failure in the recently published European Society of Cardiology (ESC) guidelines.^22^ The PRIMA II study was funded by the Dutch Heart Foundation grant 2010B97, and NT-proBNP kits were supplied by Roche Diagnostics.^18 20 21^ The authors are solely responsible for the design and conduct of this study, all study analyses, the drafting and editing of the paper and its final contents.

### Statistical analysis

Our primary endpoint for this study was 6-month all-cause mortality; the secondary endpoint was a composite endpoint of 6-month cardiovascular readmission/all cause mortality. To illustrate the relation between both endpoints (6-month all-cause mortality and 6-month cardiovascular readmission/all cause mortality) and heart failure types, we plotted the survival curve that is adjusted for clinical variables (age ≥75 years, peripheral oedema at admission, systolic blood pressure (SBP) <115 mm Hg, hyponatraemia (sodium levels <135 mmol/L) and serum urea levels ≥15 mmol/L)^11 18^ and performed the log-rank test. For statistical analysis, we used multivariable Cox regression analysis for both primary and secondary endpoints. Categorisation of absolute discharge NT-proBNP levels was done by making quartiles of NT-proBNP levels at discharge among those patients with HFpEF who died, because this creates an equal distribution of the events among the quartiles. This has the statistical advantage of creating quartiles with equal statistical power to identify predictor variables. For practical purpose, we rounded off the quartile boundaries to convenient cut-offs levels. It should be noted that for the calculation of the risk estimate and for the construction of the Kaplan Meier (KM)curves, we did use the whole population. The tertiles for NT-proBNP percentage reduction were calculated using the total cohort and were used instead of a previous dichotomous approach, because of the possible interaction with baseline NT-proBNP levels^23^ so as to discern more than ‘only’ a 30% reduction in NT-proBNP levels as prognostically relevant.

The focus of our study was to assess a possible difference in the prognostic contribution of NT-proBNP levels in patients with HFpEF versus patients with HFrEF.^22^ A separate analysis in HFmrEF was not deemed useful, as the main differences were expected between both ends of the spectrum of ejection fractions. For comparison with previous studies,^6 7^ we performed additional analyses using the 2-log and the natural logarithm scale for NT-proBNP levels at discharge for both endpoints (6-month all-cause mortality and 6-month cardiovascular readmission/all cause mortality). First, univariable Cox proportional hazard regression analysis was performed for both HFpEF and HFrEF using log-transformed NT-proBNP levels at admission and at discharge and a separate analysis using NT-proBNP quartiles at discharge. The univariable model compares the three NT-proBNP categories to the category of NT-proBNP ≤1000, therefore this is the refence category. Thereafter, we excluded the category 1001–3000 pg/mL from the multivariable model in a backward selection fashion, because it did not contain any prognostic information in our univariable model. A multivariable Cox-regression model was then made for both endpoints (6-month all-cause mortality and 6-month cardiovascular readmission/all cause mortality) with adjustment for aforementioned factors, which previously demonstrated to be prognostically significant.^11^ Similarly, HRs for relative NT-proBNP changes in categories were calculated in each of the two populations, with adjustment from the same variables. The proportionality of hazard function after transforming NT-proBNP levels was tested by generating time dependent covariates by creating interactions of the predictors and a function of survival time and including them in the model and further by testing for a non-zero slope in a generalised linear regression of the scaled Schoenfeld residuals on functions of time.^24^ Both tests supported the proportional hazards assumption.

As a measure of attainability for a number of preset NT-proBNP levels at discharge and NT-proBNP reduction percentages, the percentage of patients attaining these levels was determined for patients with HFpEF and HFrEF. Finally, the presence of comorbidities of established prognostic relevance was compared between patients with HFpEF and patients with HFrEF in a low (<3000 pg/mL) and high (>3000 pg/mL) category of discharge NT-proBNP levels. The Fisher’s exact test was used to make a comparison. Normally distributed were compared using the Student’s t-test. Other continuous data were compared using the Mann-Whitney U test.

To accommodate for the different cohorts, separate baseline hazard functions were used to adjust for between-study differences. For the multivariable model, we performed multiple imputation pooling algorithms (n=10) to correct for missing values. The imputation method was fully conditional specification with a linear regression for the model for scale variables. Eventually producing output for each ‘complete’ dataset, plus pooled output. We used these pooled results, which are pooled by Rubin’s rules, by taking the average over the parameter estimates from all imputed datasets. All patient, medical history and treatment variables (including outcome variables) were used when creating the multiple imputation data sets.

All probability values were two sided and considered significant if <0.05. Statistical analyses were conducted using SPSS V.24.0.0.0.

## Results

### Demographic characteristics

**Figure 1:**
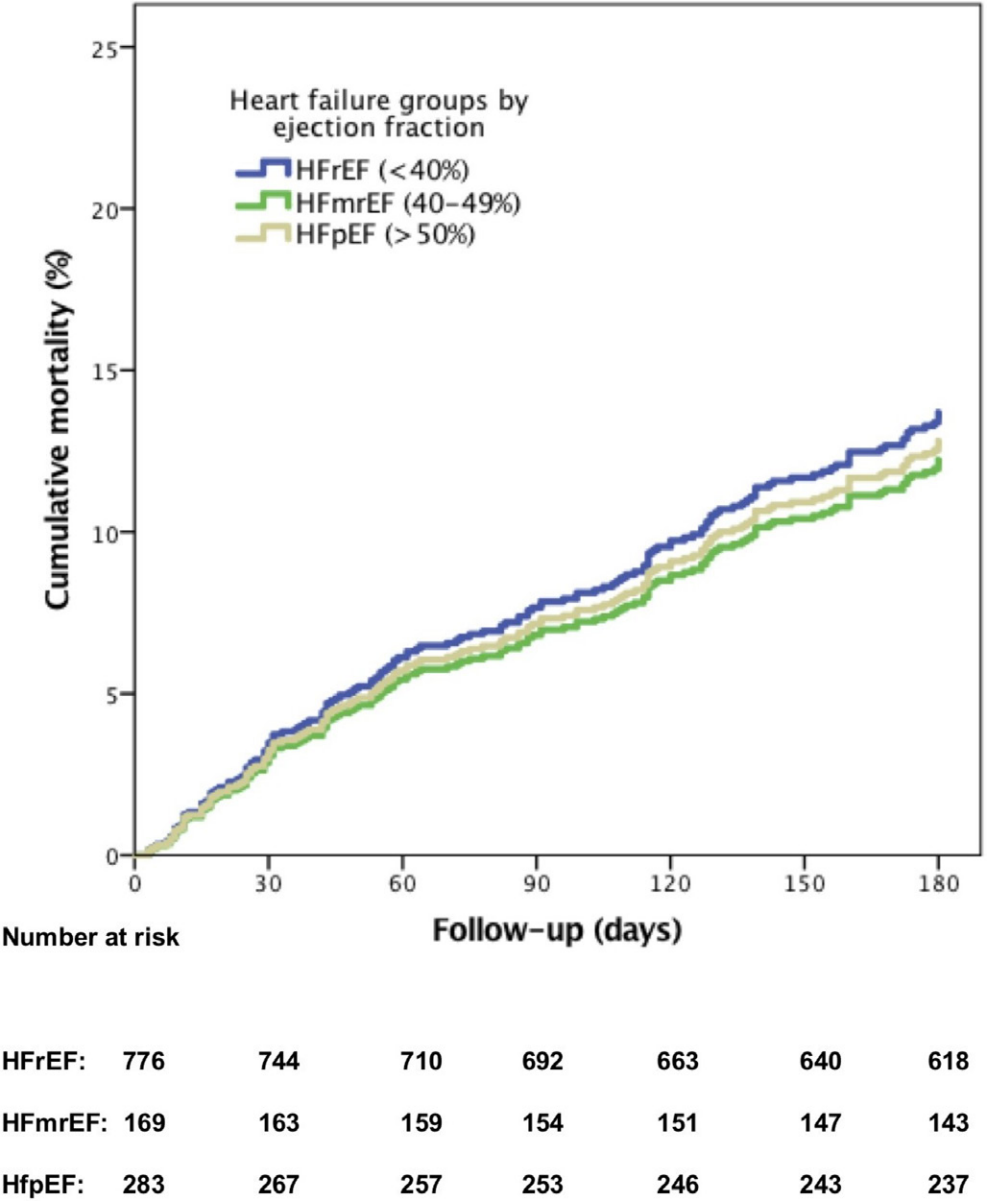
Relationship between 6-month all-cause mortality and the three types of heart failure adjusted for age ≥75 years, peripheral edema at admission, systolic blood pressure (SBP) <115 mm Hg, hyponatraemia (sodium levels <135 mmol/L) and serum urea levels ≥15 mmol/L. HFmrEF, heart failure with mid range ejection fraction; HFpEF, heart failure with preserved ejection fraction; HFrEF, heart failure with reduced ejection fraction.

Patients with HFpEF were significantly older compared with those with HFmrEF and HFrEF with significantly more patients aged ≥75 years (table 1). HFpEF was more frequently associated with female sex, hypertension, ischaemic aetiology, higher admission SBP and atrial fibrillation (AF) at admission. Compared with HFrEF and HFmrEF, HFpEF patients had significantly lower admission haemoglobin levels and lower admission and discharge NT-proBNP levels. The median NT-proBNP reduction percentage during hospitalisation was equal for HFpEF, HFmrEF and HFrEF. At discharge, significantly less HFpEF patients received ACE-inhibitors or angiotensin II receptor blockers (ARBs) compared with patients with HFmrEF and HFrEF, but diuretics and beta-blockers were prescribed with similar frequency. There was no difference in 6-month all-cause mortality (figure 1A) between patients with HFpEF, HFmrEF or HFrEF (14% (n=38) vs 14% (n=23) vs 16% (n=121), respectively, log-rank=0.60). The same pattern was seen for the composite endpoint of 6-month cardiovascular readmission/all-cause mortality (42% vs 40% vs 45%, respectively, log-rank=0.45). The median NT-proBNP percentage reduction stratified for the four NT-proBNP categories at admission for patients with HFpEF and HFrEF showed the higher the admission NT-proBNP, the higher the median percentage reduction (online supplementary materials). Supplementary figures

10.1136/heartjnl-2018-314173.supp1 Supplementary data

**Table 1:** Baseline characteristics study population

	Total cohort (n=1228)	HFpEF (≥50%) (n=283)	HFmrEF (40%–49%) (n=169)	HFrEF (<40%) (n=776)	P value
Age, years, median (IQR)	74 (64–81)	78 (70–83)	76 (70–83)	72 (61–79)	<0.001
Age ≥75 years, n (%)	596 (49)	182 (64)	96 (57)	318 (41)	<0.001
Male, n (%)	733 (60)	104 (37)	96 (57)	533 (69)	<0.001
History of DM, n (%)	376 (31)	90 (32)	61 (36)	225 (29)	0.183
History of hypertension, n (%)	659 (54)	198 (70)	99 (59)	362 (47)	<0.001
History of COPD, n (%)	206 (19)	59 (22)	32 (20)	115 (17)	0.166
Ischaemic aetiology, n (%)	548 (50)	142 (59)	77 (50)	329 (47)	0.006
Peripheral oedema at admission, n (%)	707 (62)	176 (67)	89 (61)	442 (61)	0.155
Rales at admission, n (%)	847 (75)	196 (75)	115 (78)	536 (74)	0.492
SBP at admission, mm Hg, mean±SD	135±32	145±33	141±32	130±30	<0.001
DBP at admission, mm Hg, mean±SD	81±22	81±24	79±21	81±20	0.408
Heart rate at admission, bpm, mean±SD	94±26	91±27	92±27	95±26	0.101
Atrial fibrillation at admission, n (%)	495 (43)	140 (52)	72 (46)	283 (39)	0.001
Left ventricular ejection fraction, mean±SD	36±15	58±8.8	43±2.9	26±7.0	<0.001
Laboratories findings, mean±SD					
Haemoglobin at admission, mmol/L	7.9±1.2	7.5±1.3	8.0±1.2	8.0±1.2	<0.001
Serum urea at admission, mmol/L	12±7.6	12±7.0	12±7.7	12±7.8	0.440
Serum sodium at admission, mmol/L	138±4.7	138±4.7	139±5.1	138±4.6	0.743
eGFR at admission, mL/min/1.73 m	57±33	54±24	55±28	56±35	0.611
NT-proBNP at admission, pg/mL, median (IQR)	6310 (3324–11839)	4436 (2590–8669)	5254 (3037–10868)	7173 (4039–13264)	<0.001
NT-proBNP at discharge, pg/mL, median (IQR)	3053 (1425–6661)	2147 (1114–4161)	2743 (1351–6047)	3695 (1611–7738)	<0.001
NT-proBNP reduction, %, median (IQR)	47 (20–70)	47 (25–68)	45 (17–70)	47 (19–70)	0.693
Duration admission, days, median (IQR)	9 (6–14)	7 (6–13)	8 (5–13)	9 (6–15)	0.010
Discharge medication, n (%)					
Diuretics	1076 (94)	220 (92)	144 (88)	712 (95)	0.417
ACE-inhibitor/ARBs	748 (65)	131 (55)	110 (67)	507 (68)	<0.001
Beta-blocker	744 (65)	144 (61)	105 (64)	495 (66)	0.252

ARBs, angiotensin II receptor blockers; COPD, chronic obstructive pulmonary disease; DM, diabetes mellitus; DBP, diastolic blood pressure; eGFR, estimated glomerular filtration rate; HFmrEF, heart failure with midrange ejection fraction; HFpEF, heart failure with preserved ejection fraction; HFrEF, heart failure with reduced ejection fraction; NT-proBNP, N-terminal pro-B-type natriuretic peptide; SBP, systolic blood pressure.

### NT-proBNP and outcome

**Figure 2:**
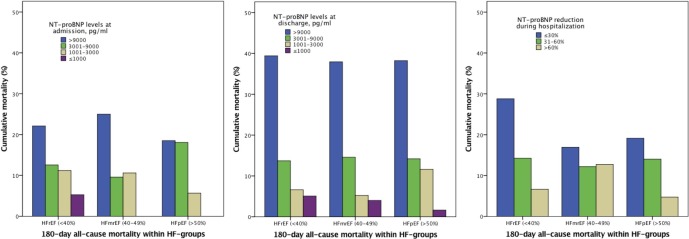
Relationship between 6-month all-cause mortality and the four categories of admission and discharge NT-proBNP levels as well as for the categories of percentage reduction during hospitalisation according to the types of HF. HF, heart failure; HFmrEF, heart failure with midrange ejection fraction; HFpEF, heart failure with preserved ejection fraction; HFrEF, heart failure with reduced ejection fraction NT-proBNP, N-terminal pro-B-type natriuretic peptide.

The lower the admission or discharge NT-proBNP levels, the lower the 6-month all-cause mortality rate (figure 2). For the three NT-proBNP percentage reduction categories, 6-month all-cause mortality decreased with higher percentage NT-proBNP reductions for all three heart failure groups.

### Cox regression models

The natural logarithmic scale for NT-proBNP levels at discharge showed that for every 2.7 increase in NT-proBNP level at discharge, the multivariably adjusted HR for 6-month mortality was 2.14 (95% CI 1.48 to 3.09) in patients with HFpEF and 1.96 (95% CI 1.60 to 2.40) in patients with HFrEF. For the composite endpoint of 6-month cardiovascular readmission/all-cause mortality, the HR was 1.27 (95% CI 1.06 to 1.52) in patients with HFpEF and 1.39 (95% CI 1.25 to 1.56) in patients with HFrEF. A log2 transformation scale for NT-proBNP levels at discharge showed an adjusted HR for 6-month mortality of 1.71 (95% CI 1.33 to 2.20) for HFpEF, and 1.60 (95% CI 1.38 to 1.99) for HFrEF for every twofold increase in NT-proBNP levels at discharge and an HR of 1.18 (95% CI 1.04 to 1.34) for HFpEF and 1.26 (95% CI 1.17 to 1.36) for the composite endpoint of 6-month cardiovascular readmission/all-cause mortality.

After adjustment for relevant clinical variables (table 2A), similar significant HRs were found for HFpEF as well as HFrEF for the highest quartile of absolute NT-proBNP values at discharge (HR 5.68, 95% CI 2.24 to 14.36 and HR 4.79, 95% CI 2.76 to 8.33, respectively). Table 2B shows that for HFpEF and HFrEF, both reduction percentage levels of 30%–60% (HR 3.28, 95% CI 1.07 to 10.12 and HR 1.79, CI 0.99 to 3.26, respectively) as well as ≤30% (HR 4.60, 95% CI 1.47 to 14.40 and HR 3.36, 95% CI 1.93 to 5.85), respectively) are predictors of mortality compared with a reference of >60%. The same pattern was seen for both the discharge NT-proBNP levels as well as percentage reduction levels and the composite endpoint of 6-month cardiovascular readmission/all-cause mortality, although with lower HRs.

**Table 2A:** Cox regression for absolute NT-proBNP levels at discharge and 6-month all-cause mortality

	HFpEF (EF ≥50%)		HFrEF (EF <40%)	
	Univariable model HR (95% CI)	Multivariable model* HR (95% CI)	Univariable model HR (95% CI)	Multivariable model* HR (95% CI)
NT-proBNP levels at discharge, pg/mL				
NT-proBNP ≤1000	1	1	1	1
NT-proBNP 1001–3000	6.59 (0.87 to 49.83)	1	1.13 (0.40 to 3.16)	1
NT-proBNP 3001–9000	7.73 (0.97 to 61.33)	1.38 (0.58 to 3.27)	2.52 (0.98 to 6.47)	1.61 (0.90 to 2.88)
NT-proBNP >9000	29.50 (3.74 to 232.87)	5.68 (2.24 to 14.36)	9.21 (3.68 to 23.00)	4.79 (2.76 to 8.33)
**Cox regression for absolute NT-proBNP levels at discharge and the composite endpoint of 6-month cardiovascular readmission/all-cause mortality**				
NT-proBNP levels at discharge, pg/mL				
NT-proBNP ≤1000	1	1	1	1
NT-proBNP 1001–3000	1.62 (0.93 to 2.85)	1	1.17 (0.75 to 1.81)	1
NT-proBNP 3001–9000	2.04 (1.13 to 3.69)	1.29 (0.82 to 2.02)	1.89 (1.26 to 2.85)	1.40 (1.06 to 1.86)
NT-proBNP >9000	3.13 (1.58 to 6.17)	1.81 (0.98 to 3.33)	3.21 (2.11 to 4.89)	2.21 (1.63 to 3.01)

*Adjusted for age ≥75 years, peripheral oedema, systolic blood pressure ≤115 mm Hg, hyponatraemia (sodium level <135 mmol/L), serum urea ≥15 mmol/L.

EF, ejection fraction; HFpEF, heart failure with preserved ejection fraction; HFrEF, heart failure with reduced ejection fraction; NT-proBNP, N-terminal pro-B-type natriuretic peptide.

**Table 2B:** Cox regression for NT-proBNP percentage reduction during hospitalisation and 6-month all-cause mortality

	HFpEF (EF ≥50%)		HFrEF (EF <40%)	
	Univariable model HR (95% CI)	Multivariable model* HR (95% CI)	Univariable model HR (95% CI)	Multivariable model* HR (95% CI)
NT-proBNP reduction during hospitalisation				
>60%	1	1	1	1
30%–60%	3.35 (1.11 to 10.11)	3.28 (1.07 to 10.12)	2.23 (1.23 to 4.04)	1.79 (0.99 to 3.26)
≤30%	5.01 (1.62 to 15.55)	4.60 (1.47 to 14.40)	5.12 (3.00 to 8.73)	3.36 (1.93 to 5.85)
**Cox regression for NT-proBNP percentage reduction during hospitalisation and the composite endpoint of 6-month cardiovascular readmission/all-cause mortality**				
NT-proBNP reduction during hospitalisation				
>60%	1	1	1	1
30%–60%	1.51 (0.91 to 2.49)	1.41 (0.83 to 2.38)	1.99 (1.48 to 2.69)	1.78 (1.32 to 2.42)
≤30%	1.63 (1.03 to 2.60)	1.69 (1.06 to 2.71)	2.77 (2.08 to 3.68)	2.21 (1.63 to 3.00)

*Adjusted for age ≥75 years, peripheral oedema, systolic blood pressure ≤115 mm Hg, hyponatraemia (sodium level <135 mmol/L), serum urea ≥15 mmol/L.

EF, ejection fraction; HFpEF, heart failure with preserved ejection fraction; HFrEF, heart failure with reduced ejection fraction; NT-proBNP, N-terminal pro-B-type natriuretic peptide.

### Attainability of NT-proBNP levels

For the lowest absolute NT-proBNP target of <1000 pg/ mL, 23% of patients with HFpEF attained the target (table 3) versus 14% of patients with HFrEF (p=0.002). Also, for the absolute level of <3000 pg/mL, patients with HFpEF more often attained this target compared with patients with HFrEF (65% vs 43%, respectively, p<0.001). No differences for attaining relative reduction levels were found between HFpEF and HFrEF. No significant differences were found between HFpEF and HFrEF in attainability of absolute or relative levels when stratified for admission NT-proBNP categories (online supplementary materials). 

**Table 3:** Attainability of absolute and relative NT-proBNP targets for types of heart failure

	Types of heart failure		
	HFpEF (EF ≥50%)	HFmrEF (40%–49%)	HFrEF (EF <40%)
Absolute NT-proBNP targets at discharge, pg/mL			
<1000, n (%)	62 (23)	25 (16)	101 (14)
<3000, n (%)	175 (65)	84 (52)	321 (43)
NT-proBNP reduction during hospitalisation, %			
>30, n (%)	187 (70)	97 (62)	483 (66)
>60, n (%)	86 (32)	55 (35)	260 (36)

EF, ejection fraction; HFmrEF, heart failure with midrange ejection fraction; HFpEF, heart failure with preserved ejection fraction; HFrEF, heart failure with reduced ejection fraction; NT-proBNP, N-terminal pro-B-type natriuretic peptide.

### NT-proBNP and comorbidities

Table 4 shows that comorbidities and prognostic factors are evenly distributed among the higher NT-proBNP discharge category in patients with HFpEF and HFrEF (61% and 60% of patients, respectively) but are unevenly distributed within the lower discharge NT-proBNP category in patients with HFpEF compared with in patients with HFrEF (37% of patients versus 24% of patients, respectively, p=0.011). There were no differences in survival between patients with HFpEF and HFrEF as compared in a low (≤3000 pg/mL) or in a high (>3000 pg/mL) NT-proBNP discharge category in KM analysis (online supplementary materials).

**Table 4:** Distribution of risk factors according to NT-proBNP levels at discharge and heart failure groups

	NT-proBNP at discharge ≤3000 pg/mL (n=580)			NT-proBNP at discharge >3000 pg/mL (n=591)		
	HFpEF	HFrEF	P value	HFpEF	HFrEF	P value
Age ≥75 years, n (%)	101 (58)	92 (29)	<0.001	64 (67)	199 (48)	0.001
Ischaemic aetiology, n (%)	97 (63)	147 (51)	0.027	43 (54)	174 (44)	0.108
Peripheral oedema at admission, n (%)	100 (63)	162 (54)	0.060	66 (73)	255 (65)	0.218
SBP ≤115 mm Hg at admission, n (%)	27 (16)	84 (26)	0.007	24 (26)	188 (45)	<0.001
Atrial fibrillation at admission, n (%)	85 (51)	110 (37)	0.005	50 (57)	162 (42)	0.017
Anaemia at admission, n (%)	77 (46)	129 (41)	0.290	59 (63)	241 (60)	0.640
Hyponatraemia (<135 mmol/L) at admission, n (%)	19 (14)	34 (11)	0.435	18 (21)	94 (23)	0.778
Serum urea ≥15 mmol/L at admission, n (%)	14 (11)	31 (11)	1.000	32 (41)	145 (39)	0.899
Comorbidities ≤2, n (%)	69 (63)	196 (76)	0.011	28 (39)	134 (40)	0.895
Comorbidities >2, n (%)	41 (37)	62 (24)		44 (61)	199 (60)	

HFpEF, heart failure with preserved ejection fraction; HFrEF, heart failure with reduced ejection fraction; NT-proBNP, N-terminal pro-B-type natriuretic peptide; SBP, systolic blood pressure.

## Discussion

It was previously reported that a doubling of natriuretic peptides at discharge carries a HR of 1.4 for (18 months) mortality, similarly for patients with HFpEF as for patients with HFrEF.^7^ In our study, for every twofold increase in discharge NT-proBNP, the multivariably adjusted HR for 6-month mortality was 1.71 in patients with HFpEF and 1.60 in patients with HFrEF, which also did not differ significantly from each other between HFpEF and HFrEF. Also, for categories, both for HFpEF as well as HFrEF, only the two highest quartiles of absolute NT-proBNP levels at discharge were predictive for mortality after adjustment for covariables, with similar HRs for patients with HFrEF as for patients with HFpEF. Our study also shows similar adjusted prognostic relative risks in patients with HFpEF and HFrEF for the relative changes in NT-proBNP levels during hospitalisation. In this respect, we confirm previous studies that have reported on equal prognostic significance of either admission^6^ levels or discharge levels of natriuretic peptides in patients with HFpEF and HFrEF hospitalised for ADHF.^7 10^

For the prognostic ability of relative changes in NT-proBNP during admissions for heart failure, we also confirm results of a small study demonstrating that changes of <30% in NT-proBNP levels during admissions are as predictive of outcome in patients with HFpEF as in a mixed population of patients with HFpEF and HFrEF.^11 12 18^ It is also in line with another report in a mixed population of patients with HFpEF and HFrEF with ADHF that described that a <50% reduction in NT-proBNP at discharge was a predictor for 6-month mortality without any significant effect from adjustment by ejection fraction.^25^ In our analysis of three categories of percentage change in NT-proBNP levels, the adjusted multivariable analysis demonstrated a significant and similar contribution to prognosis in patients with HFpEF and HFrEF for a 30%–60% reduction in NT-proBNP as well as for a ≤30% reduction in NT-proBNP. The results endorse our risk stratification model of a mixed population of patients with ADHF, in which both absolute levels at discharge and NT-proBNP reduction percentage are independent predictors of postdischarge mortality^16^ and also that both measures may be useful as discharge thresholds in patients hospitalised for ADHF.^23^

### How to interpret similar outcomes in HFpEF and HFrEF despite lower admission and discharge NT-proBNP levels in HFpEF

In our study, 6-month mortality was similar between patients with HFpEF and HFrEF, in accordance with other studies on patients with HFpEF and HFrEF after ADHF hospitalisation.^3–7^ However, like numerous other reports, we describe that natriuretic peptide levels are much lower in patients with HFpEF than in patients with HFrEF.^6 7 10 13 15 26^ The primary determinant of release of NT-proBNP is myocardial diastolic wall stress which, following the Law of LaPlace, is directly related to the transmural pressure gradient and chamber diameter and inversely related to wall thickness. Therefore, patients with HFpEF are more likely to have lower NT-proBNP levels despite a possible equally high elevated wedge pressure.^8 27^ Since the NT-proBNP levels are almost half in patients with HFpEF, the HR of 1.6–1.7 that we found for doubling NT-proBNP levels would lead to an expected reduced mortality in HFpEF with almost a factor 0.60, that is, from our reported 16% mortality in HFrEF patients to an expected 10% in patients with HFpEF. This was obviously not the case in our study, with 16% vs 14% mortality in patients with HFrEF versus patients with HFpEF after 6 months. Still, in outpatient populations and in mixed populations of outpatient and patients discharged from hospital, HFpEF patients have lower mortality rates than patients with HFrEF, which would be more in accordance with the lower NT-proBNP levels in patients with HFpEF (MAGGIC and Lam *et al* 8 9).

One of the possible answers to this paradox is that although natriuretic peptides levels are equal indicators of relative risk in patients with HFpEF and HFrEF, it is only a matter of chance finding that the outcomes of patients with HFpEF and HFrEF are alike, because other predictors of death (and possibly mode of death) and also other drivers of high NT-proBNP may be present in uneven distribution in patients with HFpEF and HFrEF,^16^ who also differ in many other baseline characteristics.

It was previously suggested that to understand prognosis in patients with HFpEF, absolute NT-proBNP levels in the lower, most frequent, categories are less contributing to prognosis in HFpEF than other prognostic variables such as kidney function or age.^28^ Also in our study, compared with patients with HFrEF, patients with HFpEF have significantly more comorbidities with significantly older patients, more frequently with hypertension, more AF at admission and lower mean levels of haemoglobin at admission, all of which were previously reported to be of prognostic significance in HFpEF but also in patients with HFrEF.^29^ Our report extends these findings, in that the presence of comorbidities probably precedes rising NT-proBNP levels more in patients with HFpEF than in patients with HFrEF, so that HFpEF patients already seem to have more comorbidities at lower NT-proBNP levels than patients with HFrEF. With higher NT-proBNP levels, the comorbidities and prognostic factors increase but are then as frequently present in HFpEF as in patients with HFrEF. To further investigate the problem of risk prediction with natriuretic peptides in the face of comorbidities, outcome differentiation and/or increasing the number of contributing comorbidities in the prognostic models both seem worthwhile.

### Attainability of NT-proBNP levels in HFpEF and HFrEF

That patients with HFpEF more often have lower NT-proBNP and BNP levels than patients with HFrEF, at similar levels of end-diastolic left ventricular pressure, is explained by the correlation of natriuretic peptide levels with diastolic wall stress, which is lower for smaller left ventricular volumes and thicker left ventricular walls, which are both hallmarks for patients with HFpEF.^27^ The similar improvements in percentage reductions in NT-proBNP in HFpEF and HFrEF may be somewhat unexpected, since there are less prognostically beneficial therapies for HFpEF than for HFpEF. At discharge, there were no significant differences in the prescription of beta-blockers or diuretics between HFpEF and HFrEF patients, while ACE inhibitors/ARBs were less often prescribed in HFpEF. We know that ARBs do not affect NT-proBNP levels in patients with HFpEF,^30^ and another explanation must be found why NT-proBNP reductions were as large in patients with HFpEF as in patients with HFrEF. The significantly higher SBP is notable among patient with HFpEF, which may have given the clinicians more opportunity to increase the dosage of diuretics during hospitalisation in HFpEF patients.

A strategy that uses absolute NT-proBNP values as risk thresholds and uses relative reductions in NT-proBNP as targets may benefit from the finding that both patient groups will be able to reach realistic but still prognostically important reductions in NT-proBNP. As for outcome, cardiovascular outcomes may be predicted by natriuretic peptides in HFpEF and HFrEF, but this study and other studies demonstrate that for patients with HFpEF with low NT-proBNP levels outcome improvements should probably also be sought in therapies targeting non-cardiovascular outcomes. Alternatively, when natriuretic peptides levels are to be used as surrogate endpoints, these endpoints should be better defined as cardiovascular endpoints.

## Limitations

Variation in NT-proBNP assays used should be considered. Nevertheless, this range in markers reflects the day-to-day clinical practice. The relatively small sample size of patients with HFpEF in the cohort compared with that of HFrEF patients should be considered, with possible impact on the statistical significance of the used NT-proBNP categories. We did correct for the bias from data missing at random by using multiple imputation pooling algorithms. The endpoint of all-cause mortality is limiting, as cardiac markers should be best used for cardiac outcomes. It is clear that CV mortality explains 63%–87% of mortality after ADHF.^31 32^ In this respect, a previous study has shown that the proportion of mortality in heart failure explained by cardiac and non-cardiac causes is the same in HFpEF as in HFrEF but maybe also because the comorbidities were more evenly distributed between HFpEF and HFrEF than in other studies.^31^

## Conclusions

Our study suggests that there is no difference between patients with HFpEF and HFrEF in relative risk prediction of 6-month mortality by absolute discharge NT-proBNP levels or by percentage NT-proBNP changes. To explain the similar long-term mortality in HFpEF as in HFrEF, despite lower NT-proBNP discharge levels in HFpEF, we raise the possibility that the larger burden of prognostic relevant comorbidities in patients with HFpEF with low NT-proBNP levels unfavourably affects their prognosis.

**Key messages**

**What is already known on this subject?**

- Prognostic information of a single measurement of natriuretic peptide levels has been specifically investigated and reported to be equal for both heart failure with preserved ejection fraction (HFpEF) and heart failure with reduced ejection fraction (HFrEF) either at admission or at discharge.

**What might this study add?**

- This study adds to the present knowledge that the percentage changes in N-terminal pro-B-type natriuretic peptide (NT-proBNP) levels during hospitalisation and absolute discharge NT-proBNP values are contributing prognostic information for 6 months without the need to distinguish between patients with HFpEF and HFrEF. Our study further highlights the possibility that comorbidities contribute relatively more to prognosis in patients with HFpEF with lower NT-proBNP levels than in patients with HFrEF with lower NT-proBNP levels. Finally, despite known treatment obstacles in patients with HFpEF, our study shows that patients with HFpEF reach similar or larger relative reductions in NT-proBNP compared with patients with HFrEF.

**How might this impact on clinical practice?**

- A strategy that uses absolute NT-proBNP values as risk thresholds and uses relative reductions in NT-proBNP as targets may benefit from the finding that both patient groups will be able to reach realistic but still prognostically important reductions in NT-proBNP. This study and other studies demonstrate that for patients with HFpEF with low NT-proBNP levels, outcome improvements should probably also be sought in therapies targeting non-cardiovascular outcomes.

10.1136/heartjnl-2018-314173.supp2 Supplementary data

10.1136/heartjnl-2018-314173.supp3 Supplementary data
