# Supplementary material for: Prognosis and NT-proBNP in heart failure patients with preserved versus reduced ejection fraction
Source: Heart. 2019 Apr 8;105(15):1182–9. doi: 10.1136/heartjnl-2018-314173 (PMC6662953; doi:10.1136/heartjnl-2018-314173)
Supplement: Supplementary data [file heartjnl-2018-314173supp001.pdf]

Supplementary material

Methods

The total cohort consisted of 1411 patients. For the present study there were no more patients excluded because of not meeting the criterium of being hospitalized because of clinically validated ADHF or because of not being discharged alive. There were 20 (1,4%) patients excluded because there were no NT-proBNP measurements available either at admission or at discharge. In addition, there were 163 (11.6%) patients excluded because there were no exact LVEF measurements available during admission. To define the extent of change in NT-proBNP during hospitalization for ADHF in HFpEF, HFmrEF and HFrEF separately, while also considering the interaction of change with baseline levels of natriuretic peptides, we calculated the median NT-proBNP percentage reduction for HFpEF, HFmrEF and HFrEF according to NT-proBNP quartiles at admission. The attainability of the absolute NT-proBNP discharge targets and relative targets (percentage reduction) was also tested after stratification in NT-proBNP quartiles at admission. Thereafter, we calculated the C-statistics (area under the receiver operating characteristic curve) for the models, with and without incorporation of NT-proBNP levels, for HFpEF and HFrEF separately. The predictive performance of NT-proBNP levels at admission, at discharge and the reduction percentage was calculated between HFpEF and HFrEF before and after adding the clinical risk markers into a model with NT-proBNP levels. The C-statistics were compared using Henley and MacNeil's method.[1]

Results

NT-proBNP reduction during hospitalization and types of HF

Supplement table 1 shows the median NT-proBNP percentage reduction stratified for the four NT-proBNP categories at admission, for HFpEF and HFrEF patients. For HFpEF and HFrEF, the higher the admission NT-proBNP, the higher the median percentage reduction.

Attainability of the NT-proBNP levels

The influence of admission NT-proBNP levels on the percentage of patients attaining different NT-proBNP targets is shown in supplement figure 1. No significant differences were found between HFpEF and HFrEF in attainability of absolute or relative levels when stratified for admission NT-proBNP categories.

NT-proBNP categories and comorbidities

There were no differences in survival between HFpEF and HFrEF patients as compared in a low (≤ 3000 pg/ml) or in a high (>3000 pg/ml) NT-proBNP discharge category in KM analysis (supplemental figure 2).

NT-proBNP levels contributing to discrimination of endpoints

Supplement table 2 shows the comparison between C-statistics of absolute NT-proBNP levels at admission and at discharge, and of the reduction percentage, before and after the addition of clinical variables for HFpEF and HFrEF. The table shows that the unadjusted C-statistics of absolute NT-proBNP levels at admission were low for both HFpEF and HFrEF (AUC 0.64, 95% CI 0.55-0.73 and AUC 0.64, 95% CI 0.59-0.69, respectively) compared to unadjusted C-statistics for NT-proBNP levels at discharge (AUC 0.70, 95% CI 0.61-0.79 and AUC 0.76, 95% CI 0.71-0.80, respectively). When adding the clinical risk markers to the models, the highest C-statistics were found for the model including NT-proBNP levels at discharge,

similarly for HFpEF (AUC 0.76, 95% CI 0.66-0.87) as well as for HFrEF (AUC 0.81, 95% CI 0.75-0.86).

Discussion

Our study shows that compared to admission levels of NT-proBNP or BNP, the discharge levels have the best reported prognostic value for HFpEF as well as for HFrEF, in line with previous studies in mixed patient populations of HFpEF and HFrEF.[2–4]

As expected, more frequent lower admission NT-proBNP values were followed by more frequent lower discharge NT-proBNP levels in HFpEF patients than in HFrEF patients. Other than that, no attainability differences were found between HFpEF and HFrEF patients.

The problem explaining why a lower discharge NT-proBNP in a HFpEF patient may carry a higher overall/ population risk than a lower NT-proBNP in a HFrEF patient, while this problem may not occur in patients with high NT-proBNP levels, because the risk level of a high NT-proBNP is already high. For the latter analysis, our data showed a non-significant higher 6-months mortality in HFpEF compared to HFrEF patients in low (≤ 3000 pg/ml) NT-proBNP discharge levels, while no difference was found between HFpEF and HFrEF patients with higher (>3000 pg/ml) NT-proBNP discharge levels.

References

1. Hanley JA, McNeil BJ. The meaning and use of the area under a receiver operating characteristic (ROC) curve. *Radiology*. 1982;143:29–36.

2. Bettencourt P. N-Terminal-Pro-Brain Natriuretic Peptide Predicts Outcome After Hospital Discharge in Heart Failure Patients. *Circulation*. 2004;110:2168–2174.

3. Bettencourt P, Azevedo A, Fonseca L, Araújo JP, Ferreira S, Almeida R, Rocha-Goncalves F, Ferreira A. Prognosis of decompensated heart failure patients with preserved systolic function is predicted by NT-proBNP variations during hospitalization. *Int J Cardiol*. 2007;117:75–79.

4. Kociol RD, Horton JR, Fonarow GC, Reyes EM, Shaw LK, O'Connor CM, Felker GM, Hernandez AF. Admission, Discharge, or Change in B-Type Natriuretic Peptide and Long-Term Outcomes: Data From Organized Program to Initiate Lifesaving Treatment in Hospitalized Patients With Heart Failure (OPTIMIZE-HF) Linked to Medicare Claims. *Circ Heart Fail*. 2011;4:628–636.
