# Supplementary material for: Prognosis and NT-proBNP in heart failure patients with preserved versus reduced ejection fraction
Source: Heart. 2019 Apr 8;105(15):1182–9. doi: 10.1136/heartjnl-2018-314173 (PMC6662953; doi:10.1136/heartjnl-2018-314173)
Supplement: Supplementary data [file heartjnl-2018-314173supp002.pdf]

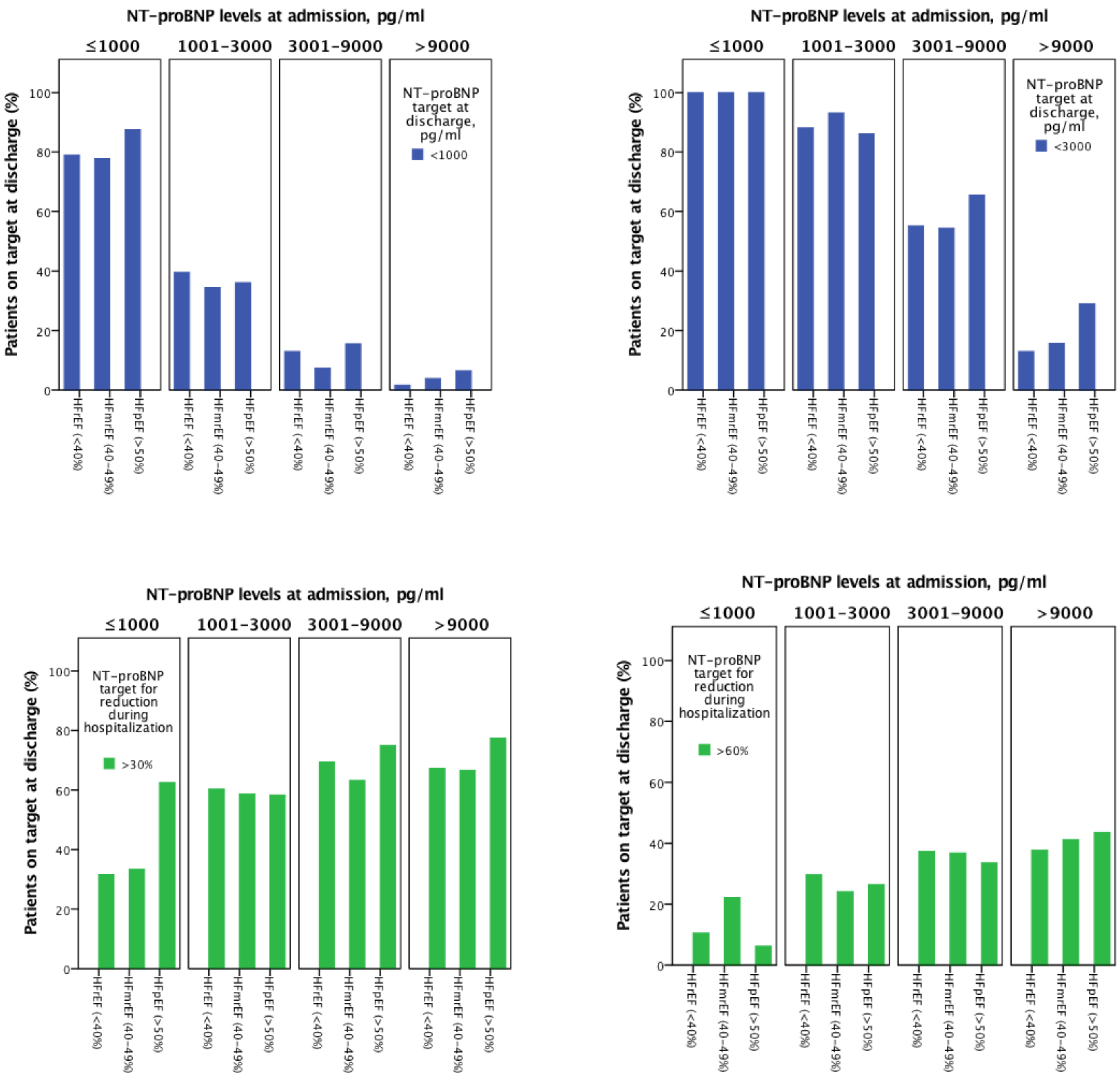

Supplement figure 1. Influence of admission NT-proBNP levels on the percentage of patients attaining different NT-proBNP targets.

Abbreviations as in figures 1 and 2.

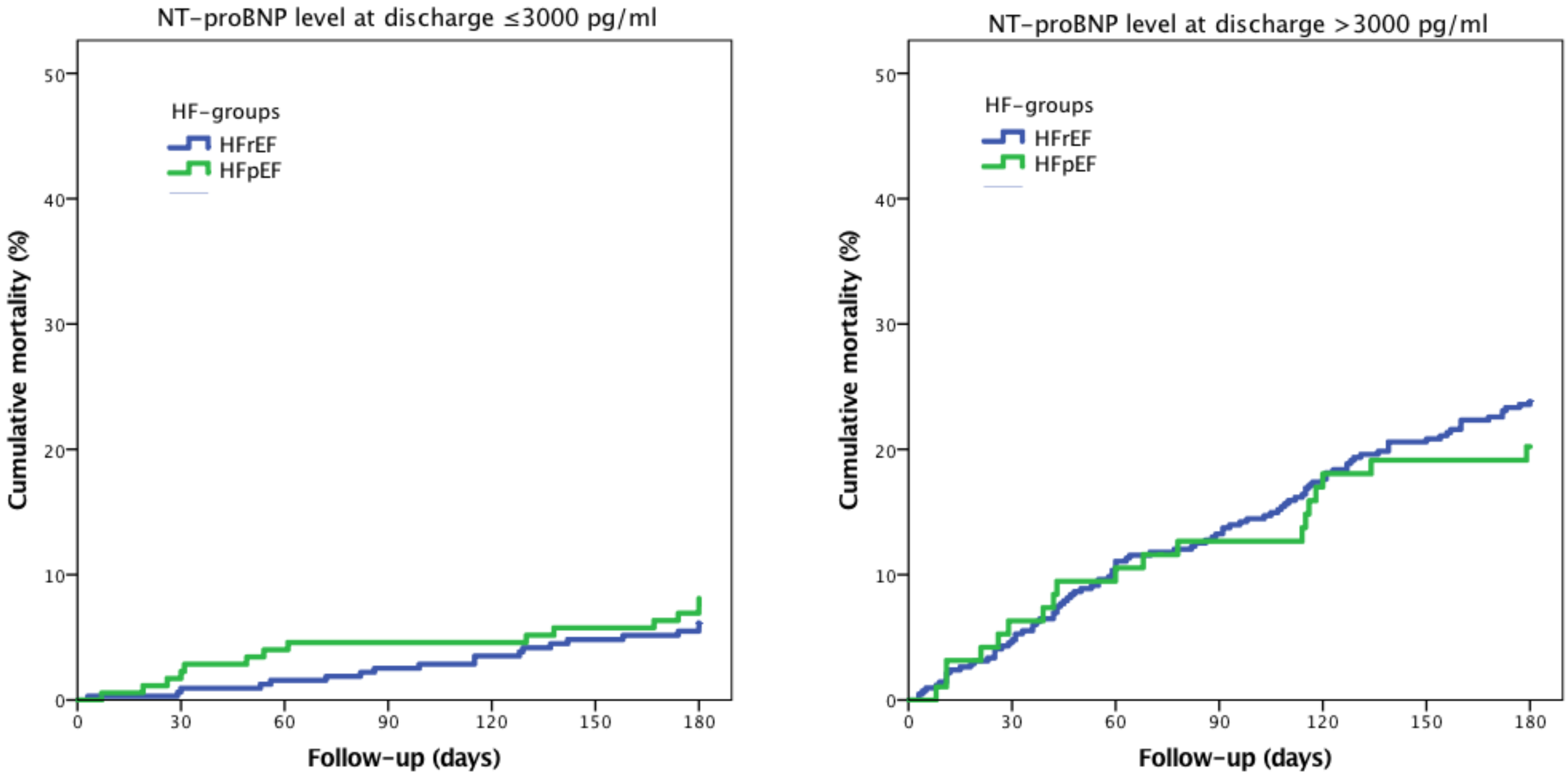

Supplement figure 2. Comparison between HF-groups according to NT-proBNP levels at discharge. Abbreviations as in figures 1 and 2.

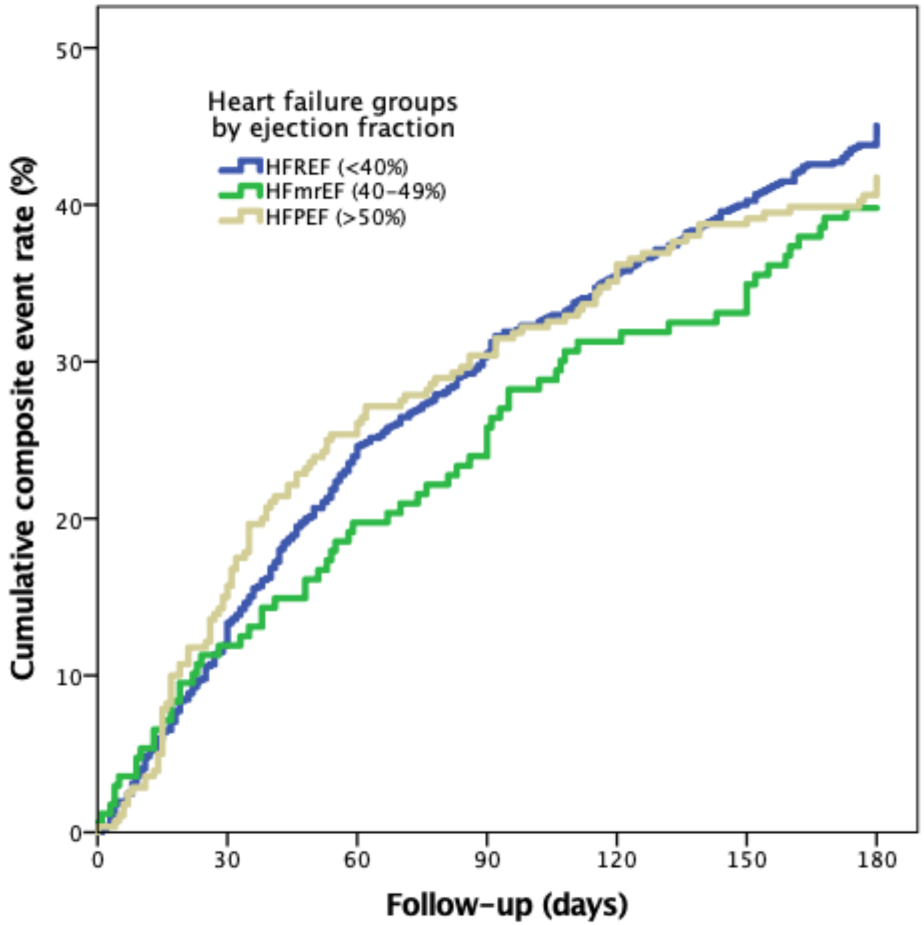

Supplement figure 3. Relationship between 6-months composite event rate (cardiovascular readmission/ all-cause mortality) and the three types of heart failure. HFpEF = heart failure with preserved ejection fraction; HFmrEF = heart failure with mid-range ejection fraction; HFrEF = heart failure with reduced ejection fraction.
