# Supplementary figures and images for: Prognosis and NT-proBNP in heart failure patients with preserved versus reduced ejection fraction
Source: Heart. 2019 Apr 8;105(15):1182–9. doi: 10.1136/heartjnl-2018-314173 (PMC6662953; doi:10.1136/heartjnl-2018-314173)

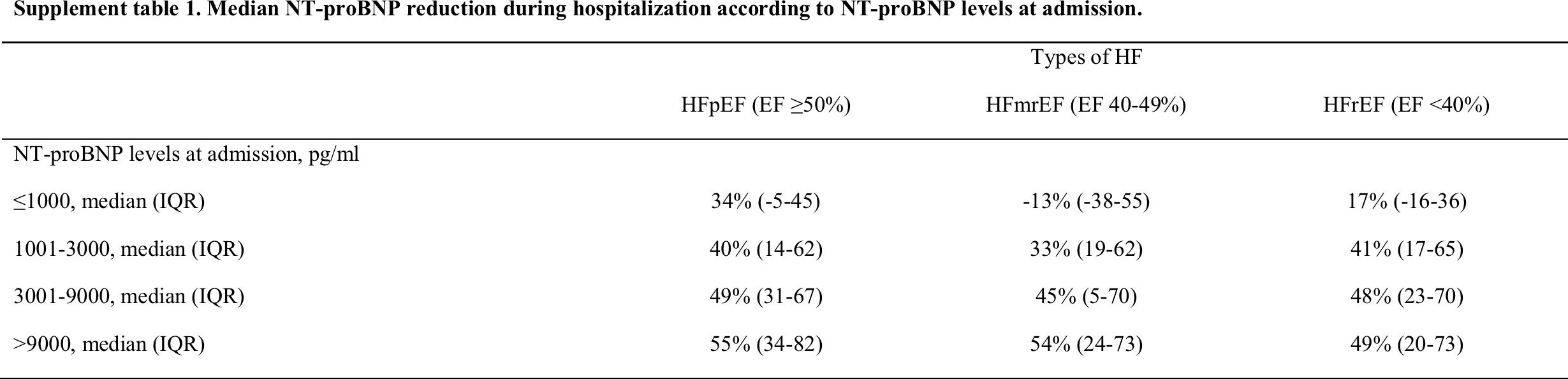

Supplement: Supplementary data [file heartjnl-2018-314173supp003.jpg]
